# A Validated Model to Predict Severe Weight Loss in Amyotrophic Lateral Sclerosis

**DOI:** 10.1002/acn3.70129

**Published:** 2025-07-08

**Authors:** David G. Lester, Kevin Talbot, Martin R. Turner, Alexander G. Thompson, the Pooled Resource Open‐Access ALS Clinical Trials Consortium

**Affiliations:** ^1^ Nuffield Department of Clinical Neurosciences University of Oxford Oxford UK

**Keywords:** amyotrophic lateral sclerosis, clinical prediction model, motor neuron disease, nutrition, weight loss

## Abstract

Severe weight loss in amyotrophic lateral sclerosis (ALS) is common, multifactorial, and associated with shortened survival. Using longitudinal weight data from over 6000 patients with ALS across three cohorts, we built an accelerated failure time model to predict the risk of future severe (≥ 10%) weight loss using five single‐timepoint clinical predictors: symptom duration, revised ALS Functional Rating Scale, site of onset, forced vital capacity, and age. Model performance and generalisability were evaluated using internal–external cross‐validation and random‐effects meta‐analysis. The overall concordance statistic was 0.71 (95% CI 0.63–0.79), and the calibration slope and intercept were 0.91 (0.69–1.13) and 0.05 (−0.11–0.21). This study highlights clinical factors most associated with severe weight loss in ALS and provides the basis for a stratification tool.

## 1 Introduction

Amyotrophic lateral sclerosis (ALS) is a fatal neurodegenerative disease characterised by progressive weakness and death, most commonly from respiratory failure [1]. Median survival from symptom onset is 30 months, and there is no highly effective disease‐modifying treatment [2]. Weight loss is a common feature of ALS, potentially driven by dietary change, dysphagia, muscle loss, respiratory weakness, and hypermetabolism [3, 4, 5]. Emerging evidence indicates metabolic dysregulation and weight loss in the years and decades before clinical presentation, suggesting a direct relationship to ALS pathobiology [6, 7]. Weight loss is associated with shortened survival in ALS, and nutritional interventions such as calorie supplementation and enteral tube feeding are cornerstones of care [8, 9, 10, 11].

The ability to predict the individual risk of future severe weight loss in people with ALS could enrich decision‐making, stratify nutrition studies, and provide insights into the drivers of weight loss. This study aimed to build and validate a prediction model based on single‐timepoint clinical measures for this purpose.

## 2 Methods

Data for model choice and feature selection were obtained from patients in the Pooled Resource Open‐Access ALS Clinical Trials (PRO‐ACT) database, comprising data from clinical trials in ALS, collected 1990–2010 and downloaded 29th July 2022 [12]. Data for model building and internal–external cross‐validation were obtained from patients in the multicentre cohort study Answer ALS, collected 2016–2019 and downloaded 13th June 2023, and the Oxford Motor Neuron Disease (MND) Centre referral‐based clinical database (Health and Social Care Northern Ireland Research Ethics Committee B Reference number 15/NI/0096), collected 2003–2019 and downloaded 7th July 2023 [13].

Statistical analysis was performed using R version 4.4.0. All three data sources were similarly processed. Inclusion criteria were ALS diagnosis and longitudinal weight data, and there were no additional exclusion criteria. Severe weight loss was defined as ≥ 10% from the first measurement, as this value amply exceeds natural weight fluctuation and has been associated with greater than two‐fold 30‐day post‐gastrostomy mortality compared to those with ALS who have lost < 10% weight [14, 15]. The potential predictor variables were those recorded at the first weight measurement: age, sex, symptom duration, site of onset, body mass index (BMI), forced vital capacity (FVC, % predicted), and revised ALS Functional Rating Scale (ALSFRS‐R). These variables were chosen as they represent many of the key single‐timepoint clinical descriptors in ALS and were available in around half or more of the cases in each of the three datasets. To minimise the risk of overfitting, the total ALSFRS‐R score was used rather than its subcomponents. Presence and timing of gastrostomy were not reliably recorded and were therefore not included as a predictor variable or censoring event.

Unrealistic outlier predictor values were removed. For the subset of patients who had ALSFRS rather than ALSFRS‐R data, question 10 (breathing) score was multiplied by three. Predicted FVC values were calculated using Global Lung Function Initiative 2012 reference values [16]. Follow‐up data were used to ascertain time to ≥ 10% weight loss or censorship.

Missingness in each of the PRO‐ACT, Answer ALS, and Oxford MND Centre datasets was inspected and then replaced with 50 imputed datasets using multivariate random forests on a dataset‐by‐dataset basis, discarding the first 50 iterations (burn‐in). All subsequent analyses were performed using Rubin's Rules to pool results from these multiply imputed datasets [17]. Predictor variable collinearity was inspected using Pearson correlation coefficients. To choose a model type, the proportionality assumption and goodness‐of‐fit of Cox proportional hazards and accelerated failure time (AFT) models fitted with all seven potential predictor variables were assessed using Schoenfeld residuals and Akaike's information criteria (AIC) in the PRO‐ACT data. Following this, feature selection was performed using fast backwards elimination in each of the 50 imputed PRO‐ACT datasets; variables that were selected more than 25 times were kept in the final model. Restricted cubic splines and interaction terms were specified a priori.

The chosen model was then fitted to the Answer ALS data and tested in the Oxford MND Centre data, and then this process was reciprocated. Test performance was measured by the concordance (*c*)‐statistic, which is the area under the receiver operating characteristic curve generalised to all possible event timepoints, and calibration slope, intercept, and curve, which reflect the numerical and graphical alignment of predicted versus observed event times. A *c*‐statistic of 1 indicates perfect pairwise event time discrimination between patients, whilst 0.5 indicates no overall stratification. A calibration slope of 1 indicates accurate predictions across risk levels, while < 1 reflects overestimated high risk and underestimated low risk predictions, and vice versa. The calibration intercept has a target value of 0; positive values suggest overall underestimated risk, and vice versa. The two sets of reciprocated discrimination and calibration performance metrics were then combined using random‐effects meta‐analysis to provide overall estimates and assessment of generalisability (internal–external cross‐validation). The model summary and nomogram were then created by fitting one model to the combined Answer ALS and Oxford MND Centre datasets.

Finally, to assess the impact of the imputation approach, a complete case‐only sensitivity analysis was performed for the full sequence described above. Findings were reported in line with the Transparent Reporting of a multivariable prediction model for Individual Prognosis or Diagnosis (TRIPOD) Statement [18].

## 3 Results

Application of inclusion criteria resulted in 5343 participants for model choice and feature selection using PRO‐ACT data and 565 and 166 participants for internal–external cross‐validation in the Answer ALS and Oxford MND Centre datasets, respectively. Patient characteristics, representing the potential predictor variables, are shown in Table 1. There were significant differences amongst the baseline predictors except for sex. A total of 1587 (29.7%), 115 (20.4%), and 53 (31.9%) participants experienced ≥ 10% weight loss during follow‐up in the PRO‐ACT, Answer ALS, and Oxford MND Centre datasets, at median times from first assessment of 8.0, 9.7, and 14.9 months, respectively. The median number of longitudinal weight measurements was 7, 4, and 3, over median follow‐up periods of 9.7, 10.6, and 10.7 months.

**TABLE 1:** Patient demographics, which represent the seven potential predictor variables.

	PRO‐ACT	Answer ALS	Oxford MND Centre	*p*
	*N* = 5343	*N* = 565	*N* = 166	
Age (years)				< 0.001
Median (IQR)	56 (16)	60 (15)	64 (16)	
Missing	98 (1.8%)	11 (1.9%)	0 (0.0%)	
Sex				0.96
Female	1971 (36.9%)	209 (37.0%)	62 (37.3%)	
Male	3274 (61.3%)	356 (63.0%)	104 (62.7%)	
Missing	98 (1.8%)	0 (0.0%)	0 (0.0%)	
Symptom duration (years)				< 0.001
Median (IQR)	1.5 (1.3)	1.8 (2.4)	1.3 (1.4)	
Missing	448 (8.4%)	11 (1.9%)	4 (2.4%)	
Site of onset				
Bulbar	720 (13.5%)	151 (26.7%)	44 (26.5%)	< 0.001
Non‐bulbar	2760 (51.7%)	394 (69.7%)	111 (66.9%)	
Missing	1863 (34.9%)	20 (3.5%)	11 (6.6%)	
Body mass index (kg/m^2^)				< 0.001
Median (IQR)	25.3 (5.7)	26.3 (6.3)	25.1 (4.8)	
Missing	0 (0.0%)	5 (0.9%)	0 (0.0%)	
Forced vital capacity (%, predicted)				< 0.001
Median (IQR)	84.2 (26.1)	79.5 (29.0)	89.5 (32.0)	
Missing	2828 (52.9%)	315 (55.8%)	10 (6.0%)	
ALSFRS‐R				< 0.001
Median (IQR)	38 (8)	36 (11)	43 (7)	
Missing	1175 (22.0%)	12 (2.1%)	41 (24.7%)	

*Note: p* values calculated using chi‐square and Kruskal‐Wallis tests for categorical and continuous variables.

Abbreviations: ALS, amyotrophic lateral sclerosis; ALSFRS‐R, revised ALS Functional Rating Scale; IQR, interquartile range; kg/m^2^, kilograms per metre squared; MND, motor neuron disease; PRO‐ACT, Pooled Resource Open‐Access ALS Clinical Trials.

Inspection of missing data before imputation shows general patterns in all three cohorts (Figure S1), and collinearity between predictor variables was as anticipated (Figure S2). Three of the seven potential baseline predictors did not meet the Cox proportional hazards assumption. Therefore, an AFT model was chosen, and a log‐logistic distribution was selected based on the lowest AIC compared to Weibull, logistic, log‐normal, and exponential distributions.

**FIGURE 1:**
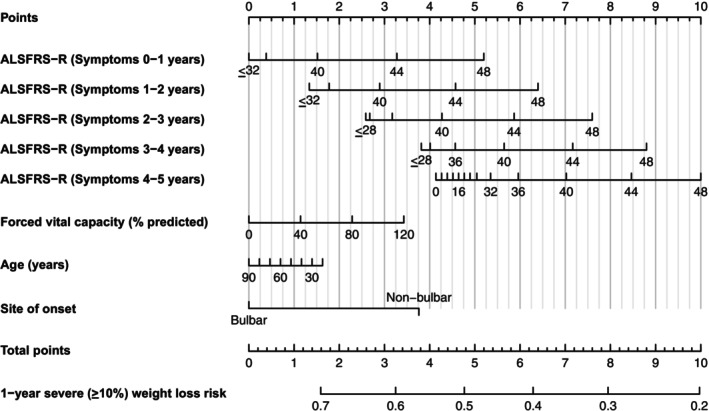
Nomogram for predicting the risk of severe (≥ 10%) weight loss over the following year in people with amyotrophic lateral sclerosis (ALS). Points for each variable are totalled using the scale at the top. The chosen row for ALSFRS‐R should correspond to the patient's symptom duration. ALSFRS‐R, revised ALS Functional Rating Scale.

Five predictor variables were selected for inclusion in the final model following backwards elimination in each of the 50 imputed PRO‐ACT datasets: age, ALSFRS‐R, site of onset, FVC, and symptom duration, all of which were selected in all 50 models. Sex and BMI were not selected in any of the imputed PRO‐ACT datasets. To capture non‐linear effects, ALSFRS‐R was modelled using restricted cubic splines with three knots, and an interaction term was specified between ALSFRS‐R and symptom duration. The summary of the combined AFT model fitted over both the Answer ALS and Oxford MND datasets is shown in Table 2, and a nomogram in Figure 1.

**TABLE 2:** Model summary.

	Estimate (95% CI)	Standardised estimate (95% CI)	Standard error	*p*
Intercept	5.94 (5.63–6.25)	6.39 (6.33–6.45)	0.598	< 0.001
Age (years)	−0.0026 (−0.0042 to −0.0010)	−0.032 (−0.050 to −0.014)	0.00389	0.51
Forced vital capacity (%, predicted)	0.003 (−0.0004 to 0.0066)	0.050 (−0.030 to 0.130)	0.00276	0.26
Site of onset (non‐bulbar)	0.411 (0.354 to 0.468)	0.185 (0.159 to 0.210)	0.0925	< 0.001
Symptom duration (years)	0.032 (−0.036 to 0.101)	0.511 (0.418 to 0.604)	0.129	0.80
ALSFRS‐R spline 1	−0.012 (−0.022 to −0.00089)	0.015 (−0.070 to 0.100)	0.0192	0.55
ALSFRS‐R spline 2	0.032 (0.021 to 0.0425)	0.159 (0.073 to 0.245)	0.0191	0.10
Interaction (symptom duration:ALSFRS‐R spline 1)	0.0037 (0.00073 to 0.0066)	0.093 (0.066 to 0.121)	0.00484	0.45
Interaction (symptom duration:ALSFRS‐R spline 2)	−0.0025 (−0.0084 to 0.0033)	−0.064 (−0.199 to 0.072)	0.00766	0.741

*Note:* Shown additionally with standardised estimates (z‐score normalised, mean = 0, standard deviation = 1).

Abbreviations: ALSFRS‐R, revised ALS Functional Rating Scale; CI, confidence interval.

**FIGURE 2:**
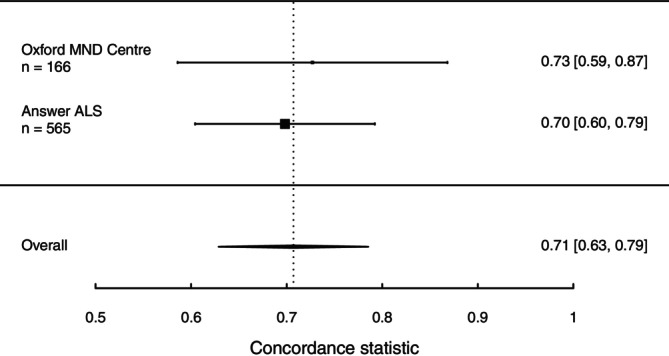
Forest plot of the concordance statistics for a model trained on Answer ALS data and tested on Oxford MND Centre data (top), vice versa (middle), and the overall performance from a random‐effects meta‐analysis of the two reciprocated results (bottom). ALS, amyotrophic lateral sclerosis; MND, motor neurone disease.

**FIGURE 3:**
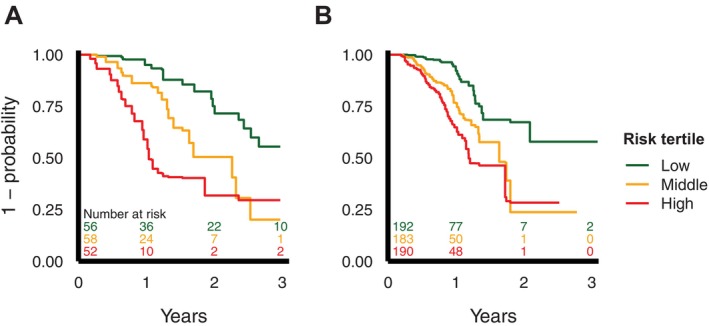
Kaplan–Meier curves representing the cumulative probability of not experiencing severe (≥ 10%) weight loss over time for patients in model‐defined risk tertiles. Represented as (A) Oxford MND Centre patients divided into risk tertiles as defined by a model trained on Answer ALS data and (B) Answer ALS patients divided into risk tertiles as defined by a model trained on Oxford MND Centre data. Risk tables are inset. ALS, amyotrophic lateral sclerosis; MND, motor neurone disease.

Internal–external cross‐validation and random‐effects meta‐analysis provided a good overall *c*‐statistic of 0.71 (95% CI 0.63–0.79) with consistency across the datasets (Figure 2). Discriminatory performance of the model fitted in the Answer ALS data and tested in the Oxford MND Centre data, and vice versa, was visualised using Kaplan–Meier curves of patients divided into their predicted risk tertiles (Figure 3A,B). Calibration curves were satisfactory on inspection (Figure S3A,B), and overall estimates for calibration slope and intercept for one‐year predictions were good at 0.91 (0.69–1.13) and 0.05 (−0.11–0.21), respectively (Figure S3C,D), with some heterogeneity between the datasets.

In the complete case‐only sensitivity analysis, a Cox model was chosen over an AFT model based on lower AIC and satisfactory proportional hazards assumption for all seven potential predictor variables in the 1258 complete cases present in the PRO‐ACT dataset. Fast backwards elimination using AIC identified age, ALSFRS‐R, FVC, and symptom duration as predictor variables. A three‐knotted restricted cubic spline was applied to ALSFRS‐R, and an interaction term was specified between ALSFRS‐R and symptom duration. Internal–external cross‐validation and random‐effects meta‐analysis of Cox models fitted with these four predictors in the 236 and 108 complete cases in the Answer ALS and Oxford MND Centre datasets produced a poor overall *c*‐statistic of 0.27 (0.16–0.37) and suboptimal calibration curves and one‐year slopes and intercepts (Figure S4A–D).

## 4 Discussion

Using longitudinal weight data collected from over 6000 people with ALS across three heterogeneous cohorts, we have built and validated a model that accurately predicts the risk of future severe weight loss using single‐timepoint clinical variables. Generalisability across cohorts is demonstrated, despite differences in baseline demographics. The model could be updated using new cohorts. A nomogram and model summary are provided, which may be used to generate individualised predictions. We show that sex and BMI lack multivariable predictive power for severe weight loss in ALS, unlike previous work using PRO‐ACT data [19]. Greatest risks are seen in those with reduced ALSFRS‐R in conjunction with shorter symptom duration, bulbar onset, reduced FVC, and increased age. This is in keeping with established associations between disease aggressiveness, diagnostic latency, and severe weight loss; we note that this model's variables are a subset of those in the ENCALS survival prediction model [10, 20].

Strengths of this study are the use of the large PRO‐ACT dataset for model choice and feature selection, multiple imputation to preserve partial information, and the use of internal–external cross‐validation to understand the generalisability of a model built across clinical cohorts. The principal limitation is the lack of data for additional factors that may influence the risk of weight loss that therefore could not be included as predictor variables, such as prediagnostic weight loss, metabolic comorbidities, and metabolic measurements, for example, calorimetry, body composition, or biomarkers of lipid and carbohydrate metabolism [21]. Further, the study does not account for the influence of gastrostomy insertion and nutritional supplementation on the risk of weight loss.

## Author Contributions

D.G.L. and A.G.T. contributed to the conception, methodology and analysis of the manuscript. D.G.L. drafted the original manuscript, and D.G.L., K.T., M.R.T. and A.G.T. contributed to the review and editing of the manuscript.

## Conflicts of Interest

The authors declare no conflicts of interest related to this study.

## Supporting information


**Data S1.** **Figure S1.** Missingness patterns in the (A) PRO‐ACT, (B) Answer ALS and (C) Oxford MND Centre datasets’ predictors variables. Each row represents a pattern of missingness, and row numbers indicate total occurrences of that specific pattern. Red = missing. Blue = present. Vertical totals equate to missingness; for example, there are 71+27 = 98 instances of missing age value in PRO‐ACT. ALSFRS‐R, revised ALS Functional Rating Scale; PRO‐ACT, Pooled Resource Open‐Access ALS Clinical Trials; ALS, amyotrophic lateral sclerosis; MND, motor neuron disease.
**Figure S2.** Overall predictor variable pairwise Pearson correlations after multiple imputation in the (A) PRO‐ACT, (B) Answer ALS and (C) Oxford MND Centre datasets. PRO‐ACT, Pooled Resource Open‐Access ALS Clinical Trials; ALS, amyotrophic lateral sclerosis; MND, motor neuron disease; ALSFRS‐R, revised ALS Functional Rating Scale.
**Figure S3.** Overall calibration curves calculated using Cox‐Snell residuals to evaluate the agreement between predicted and observed event rates, for (A) an accelerated failure time (AFT) model trained in Oxford MND Centre and tested in Answer ALS data, and (B) an AFT model trained in Oxford MND Centre data and tested in Answer ALS data. (C) and (D) represent the internal–external cross‐validation and random‐effects meta‐analysis overall estimates of calibration slope and intercept for one‐year predictions. ALS, amyotrophic lateral sclerosis; MND, motor neuron disease.
**Figure S4.** Complete case‐only sensitivity analysis overall calibration curves calculated using Cox‐Snell residuals to evaluate the agreement between predicted and observed event rates, for (A) a Cox model trained in Oxford MND Centre data and tested in Answer ALS data, and (B) a Cox model trained in Answer ALS data and tested in Oxford MND Centre data. (C) and (D) show calibration plots, slopes and intercepts for one‐year predictions using the same models as (A) and (B), respectively. ALS, amyotrophic lateral sclerosis; MND, motor neuron disease.

## Data Availability

Data from the PRO‐ACT database, Answer ALS and Oxford MND Centre are available to researchers following a successful registration and application process.
