# Supplementary material for: A Validated Model to Predict Severe Weight Loss in Amyotrophic Lateral Sclerosis
Source: Ann Clin Transl Neurol. 2025 Jul 8;12(9):1907–12. doi: 10.1002/acn3.70129 (PMC12455886; doi:10.1002/acn3.70129)
Supplement: Supplementary file 1 — Data S1. Figure S1. Missingness patterns in the (A) PRO‐ACT, (B) Answer ALS and (C) Oxford MND Centre datasets’ predictors variables. Each row represents a pattern of missingness, and row numbers indicate total occurrences of that specific pattern. Red = missing. Blue = present. Vertical totals equate to missingness; for example, there are 71+27 = 98 instances of missing age value in PRO‐ACT. ALSFRS‐R, revised ALS Functional Rating Scale; PRO‐ACT, Pooled Resource Open‐Access ALS Clinical Trials; ALS, amyotrophic lateral sclerosis; MND, motor neuron disease. Figure S2. Overall predictor variable pairwise Pearson correlations after multiple imputation in the (A) PRO‐ACT, (B) Answer ALS and (C) Oxford MND Centre datasets. PRO‐ACT, Pooled Resource Open‐Access ALS Clinical Trials; ALS, amyotrophic lateral sclerosis; MND, motor neuron disease; ALSFRS‐R, revised ALS Functional Rating Scale. Figure S3. Overall calibration curves calculated using Cox‐Snell residuals to evaluate the agreement between predicted and observed event rates, for (A) an accelerated failure time (AFT) model trained in Oxford MND Centre and tested in Answer ALS data, and (B) an AFT model trained in Oxford MND Centre data and tested in Answer ALS data. (C) and (D) represent the internal–external cross‐validation and random‐effects meta‐analysis overall estimates of calibration slope and intercept for one‐year predictions. ALS, amyotrophic lateral sclerosis; MND, motor neuron disease. Figure S4. Complete case‐only sensitivity analysis overall calibration curves calculated using Cox‐Snell residuals to evaluate the agreement between predicted and observed event rates, for (A) a Cox model trained in Oxford MND Centre data and tested in Answer ALS data, and (B) a Cox model trained in Answer ALS data and tested in Oxford MND Centre data. (C) and (D) show calibration plots, slopes and intercepts for one‐year predictions using the same models as (A) and (B), respectively. ALS, amyotrophic lateral sclerosis; MN [file ACN3-12-1907-s001.docx]

**Supplementary data**

**
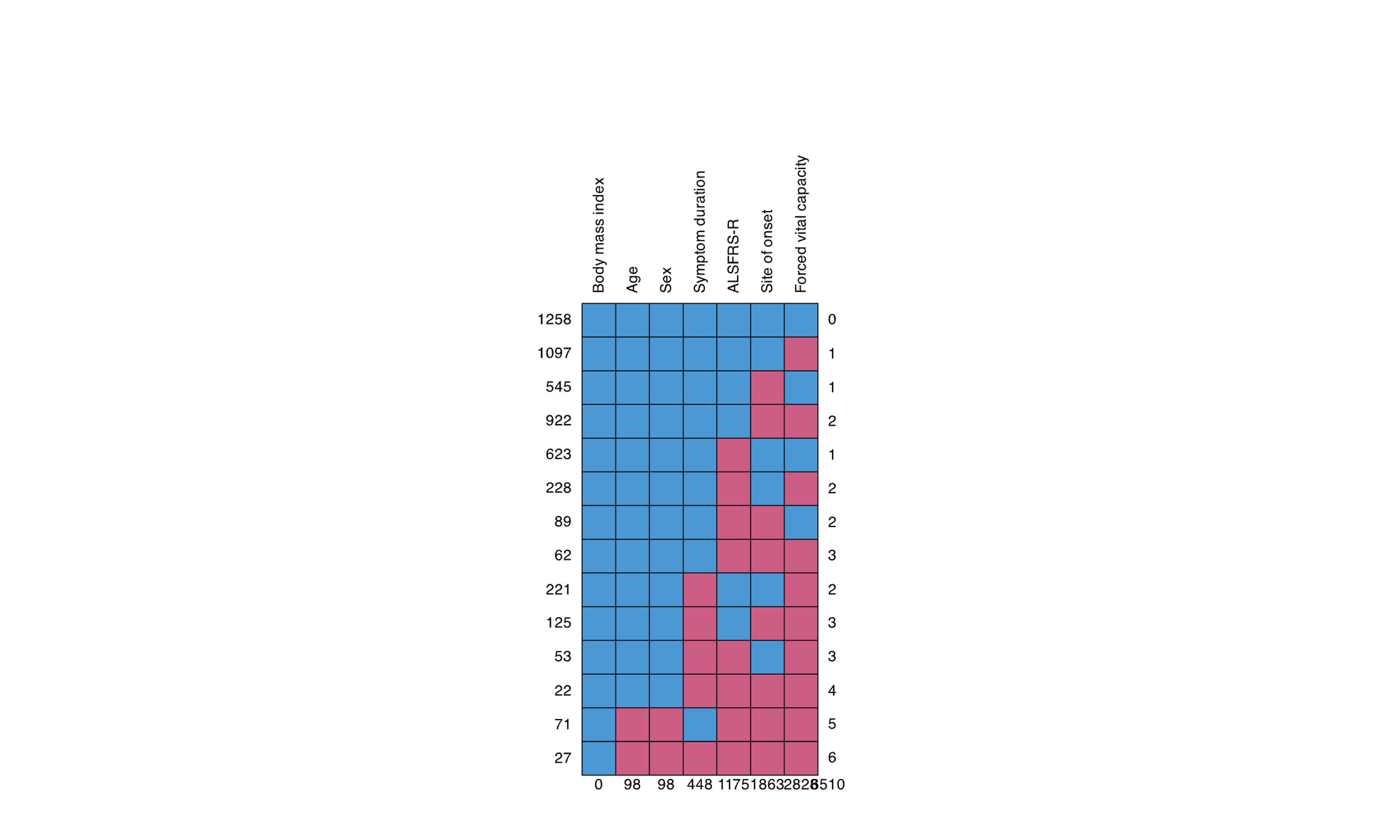
A B C**

**
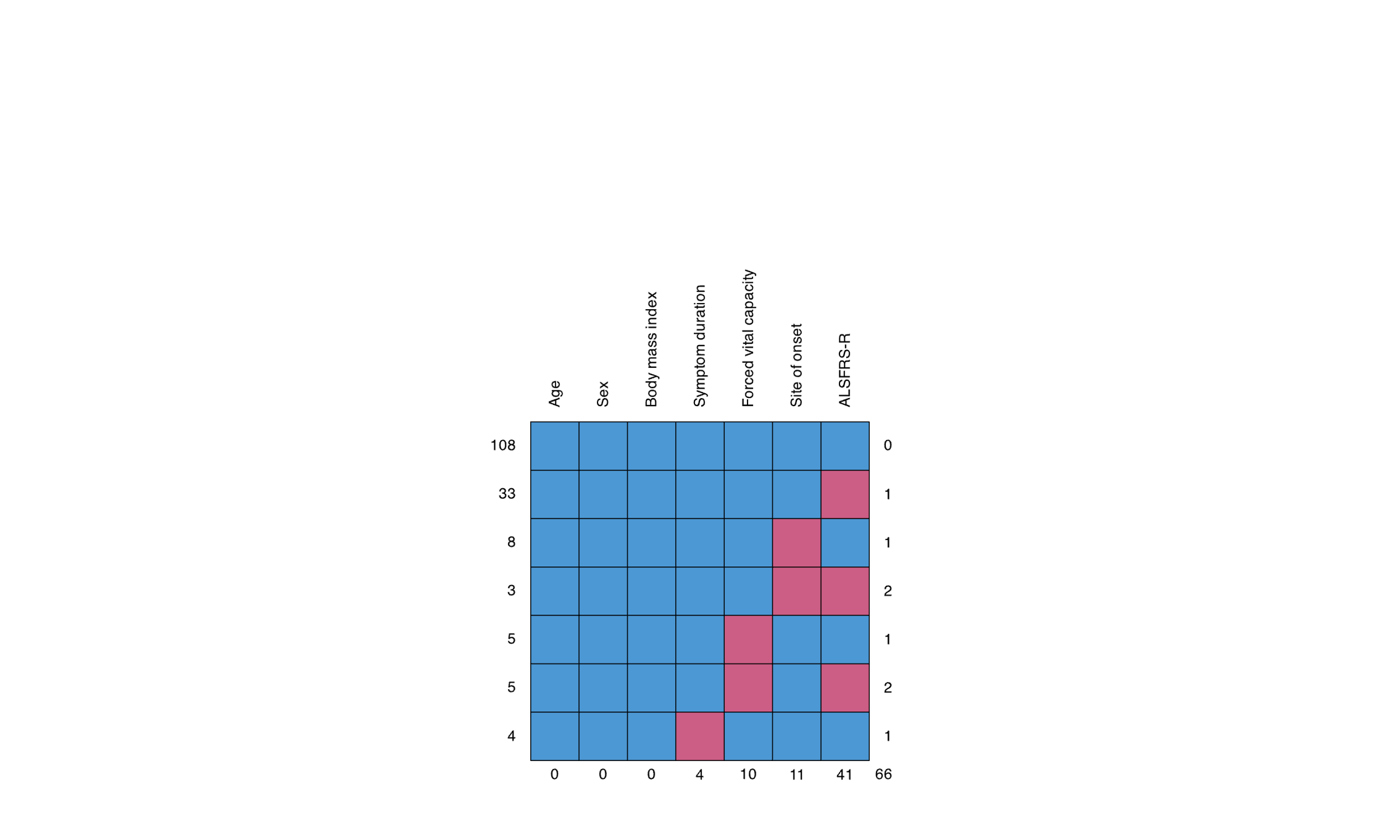

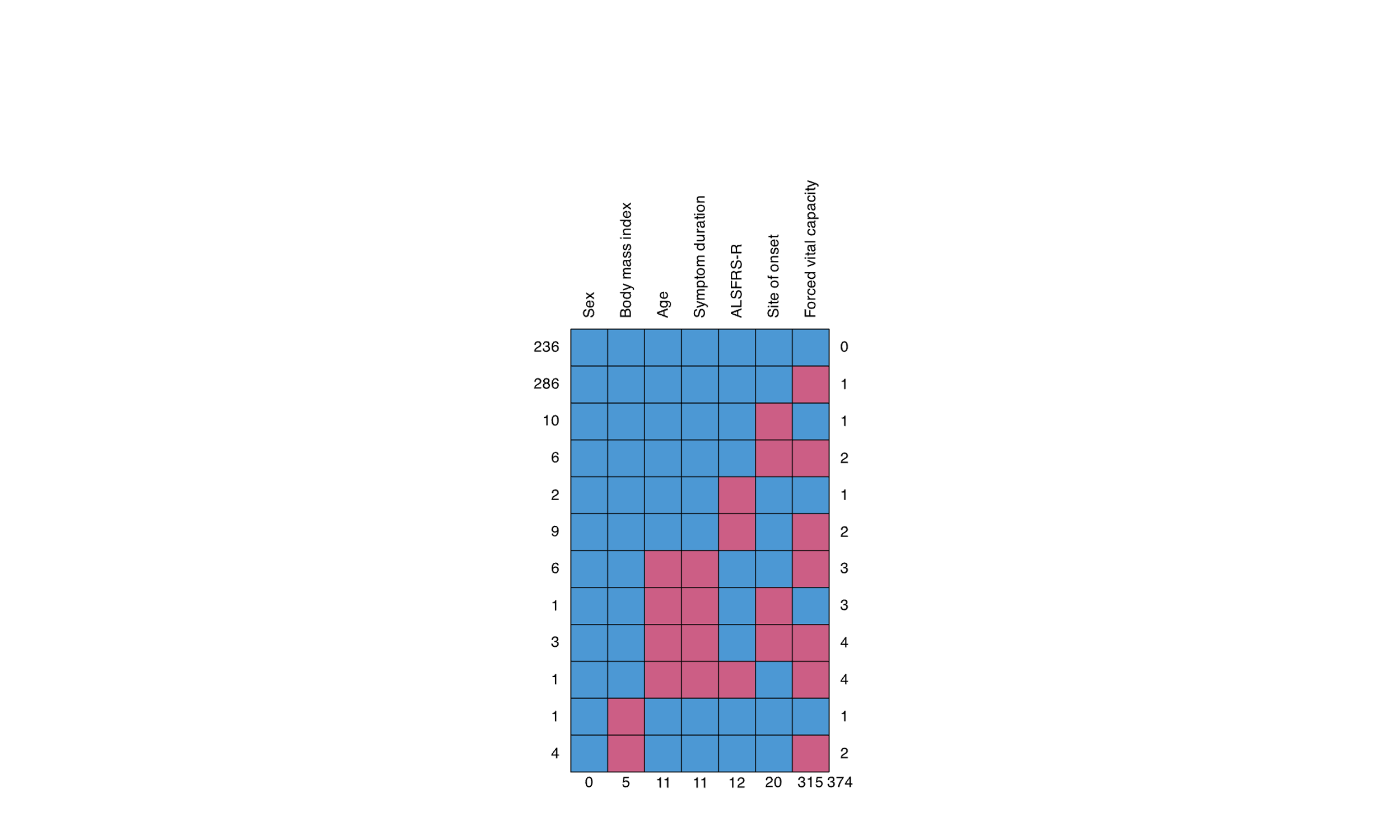
**

Supplementary figure 1. Missingness patterns in the A) PRO-ACT, B) Answer ALS and C) Oxford MND Centre datasets’ predictors variables. Each row represents a pattern of missingness, and row numbers indicate total occurrences of that specific pattern. Red=missing. Blue=present. Vertical totals equate to missingness; for example, there are 71+27=98 instances of missing age value in PRO-ACT. ALSFRS-R, revised ALS Functional Rating Scale; PRO-ACT, Pooled Resource Open-Access ALS Clinical Trials; ALS, amyotrophic lateral sclerosis; MND, motor neuron disease.

**A B**

**
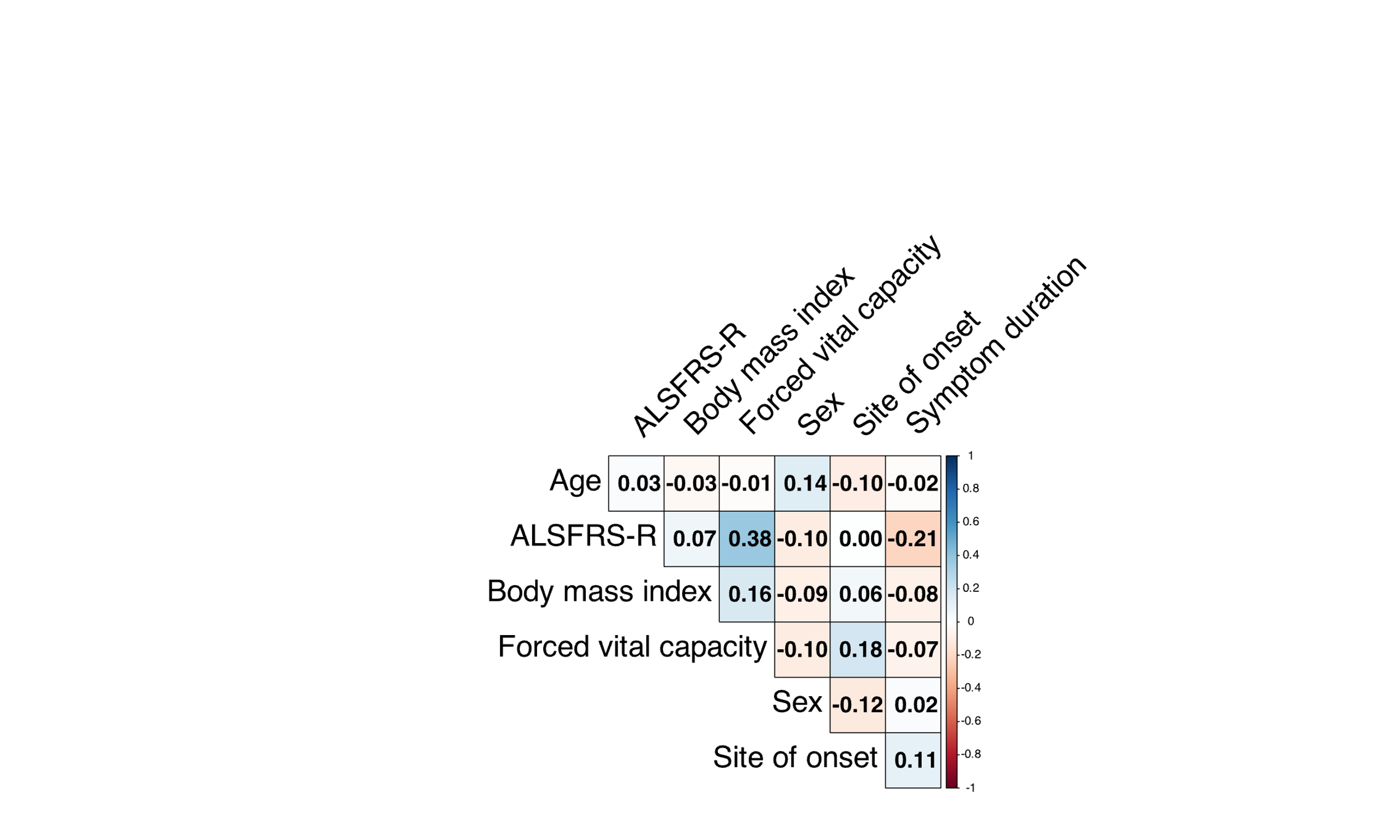

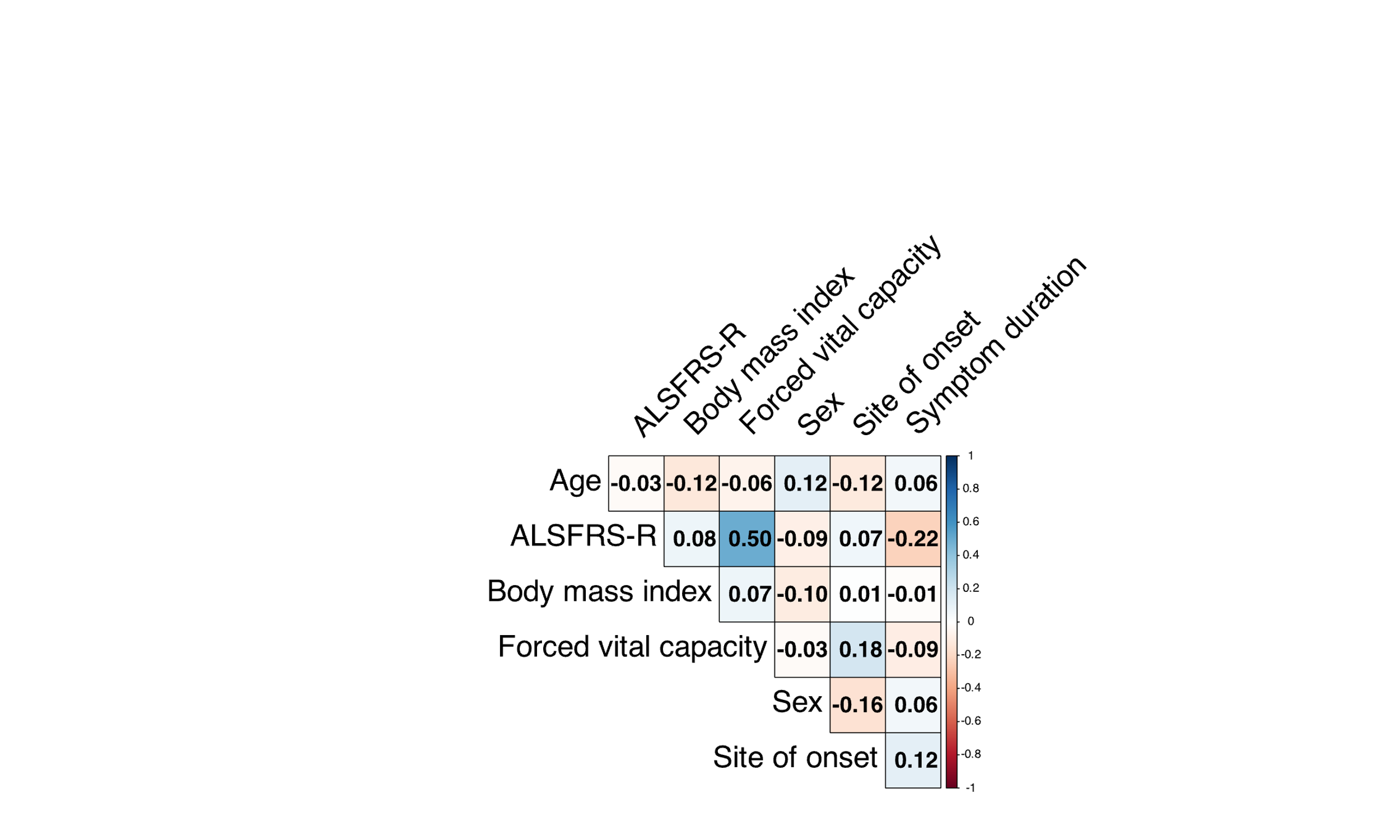
**

**C**


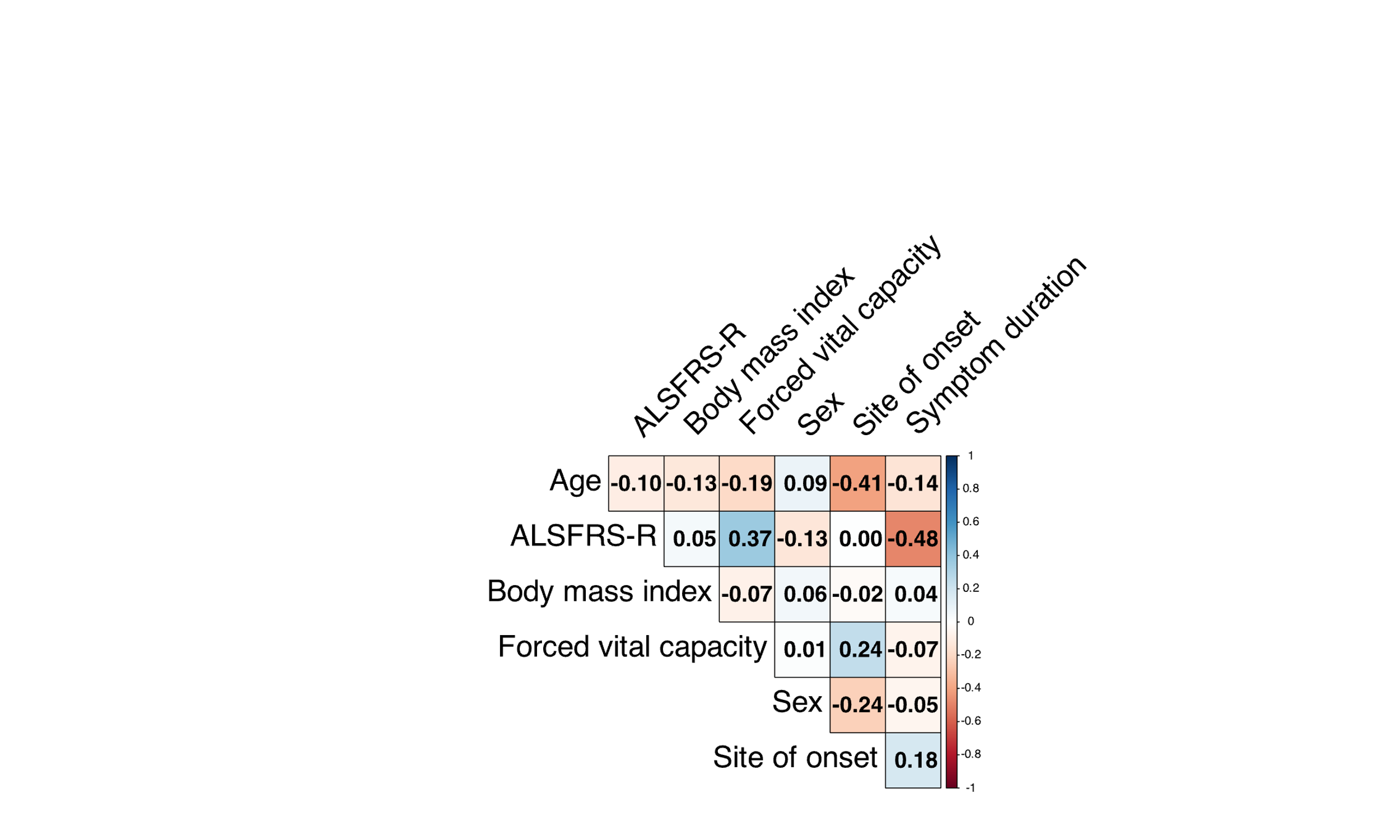


Supplementary figure 2. Overall predictor variable pairwise Pearson correlations after multiple imputation in the A) PRO-ACT, B) Answer ALS and C) Oxford MND Centre datasets. PRO-ACT, Pooled Resource Open-Access ALS Clinical Trials; ALS, amyotrophic lateral sclerosis; MND, motor neuron disease; ALSFRS-R, revised ALS Functional Rating Scale.

**A B**


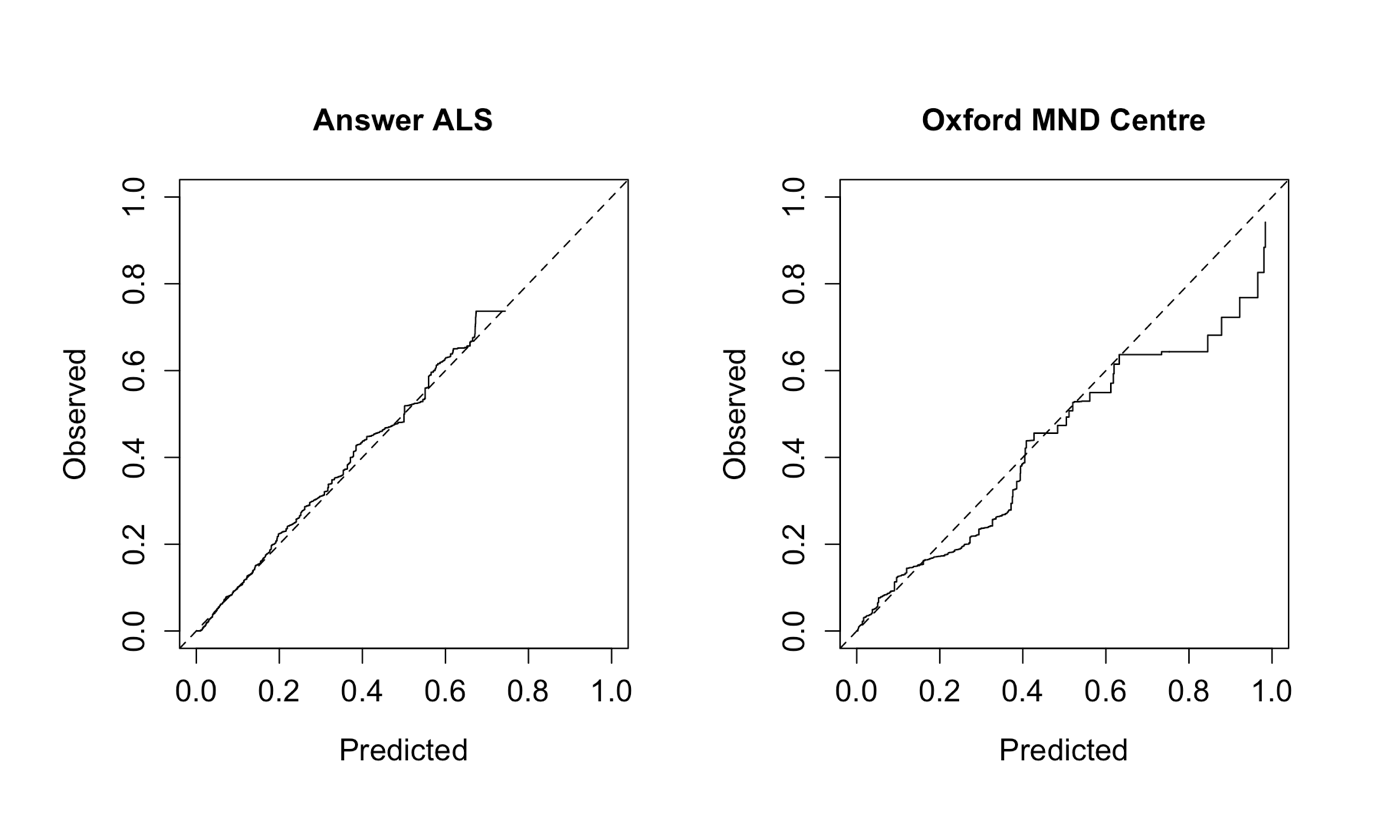


**C D**

**
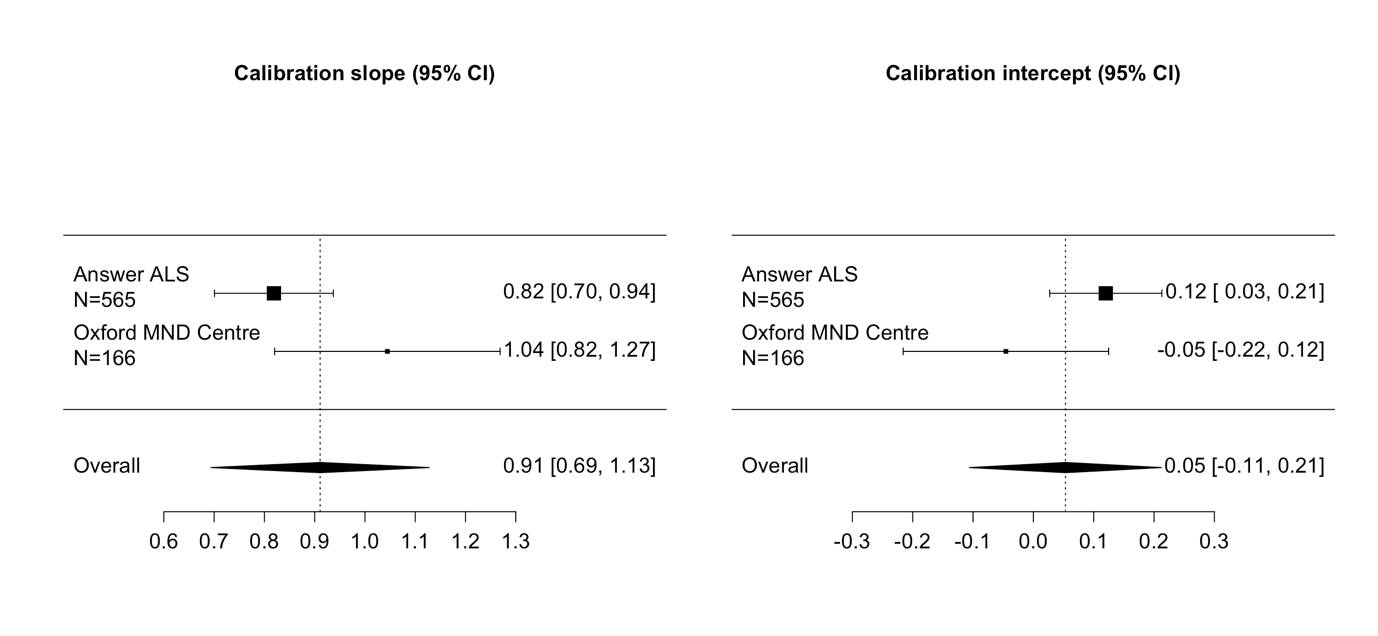
**

Supplementary figure 3. Overall calibration curves calculated using Cox-Snell residuals to evaluate the agreement between predicted and observed event rates, for (A) an accelerated failure time (AFT) model trained in Oxford MND Centre and tested in Answer ALS data, and (B) an AFT model trained in Oxford MND Centre data and tested in Answer ALS data. (C) and (D) represent the internal-external cross-validation and random-effects meta-analysis overall estimates of calibration slope and intercept for one-year predictions. ALS, amyotrophic lateral sclerosis; MND, motor neuron disease.

**A B**

**
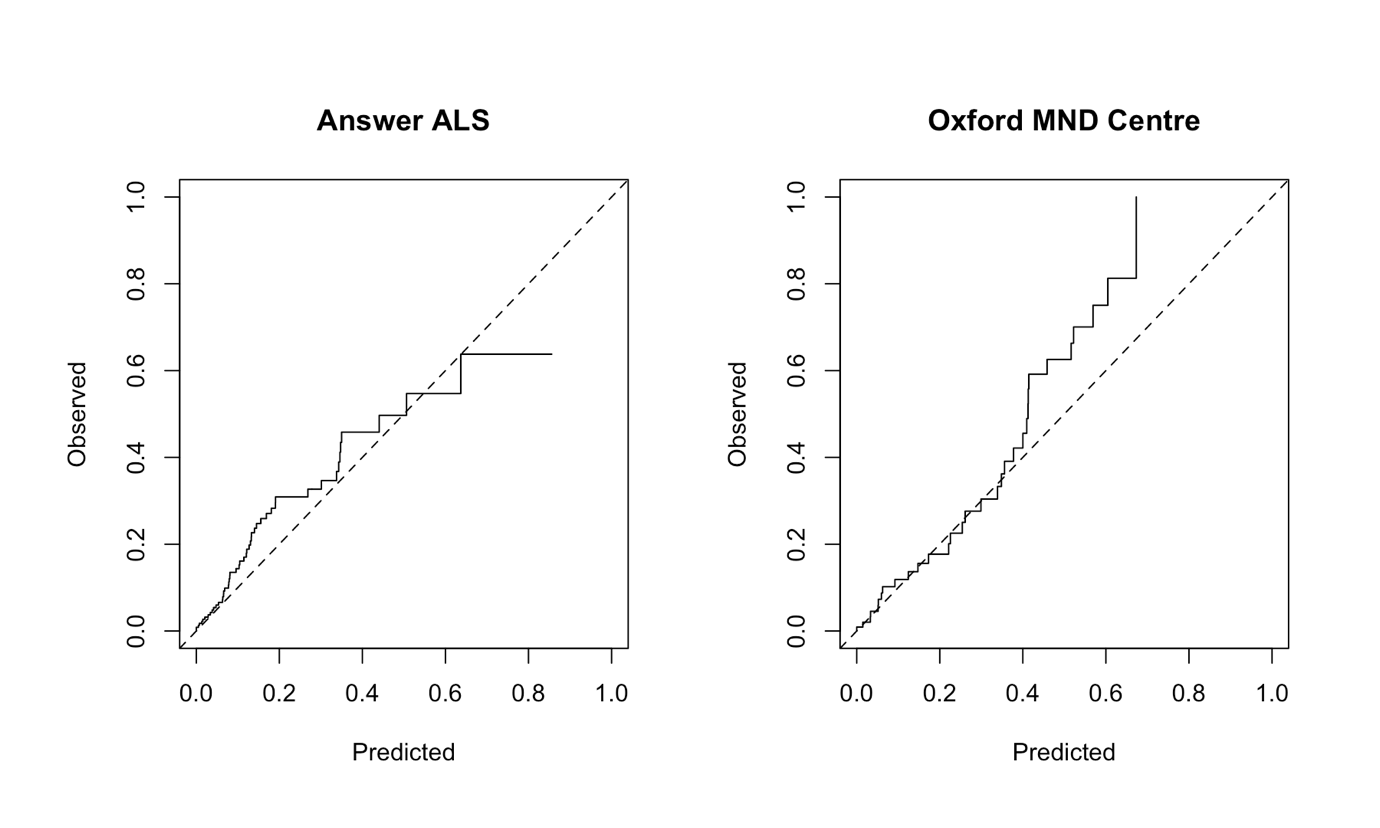
**

**C D**

**
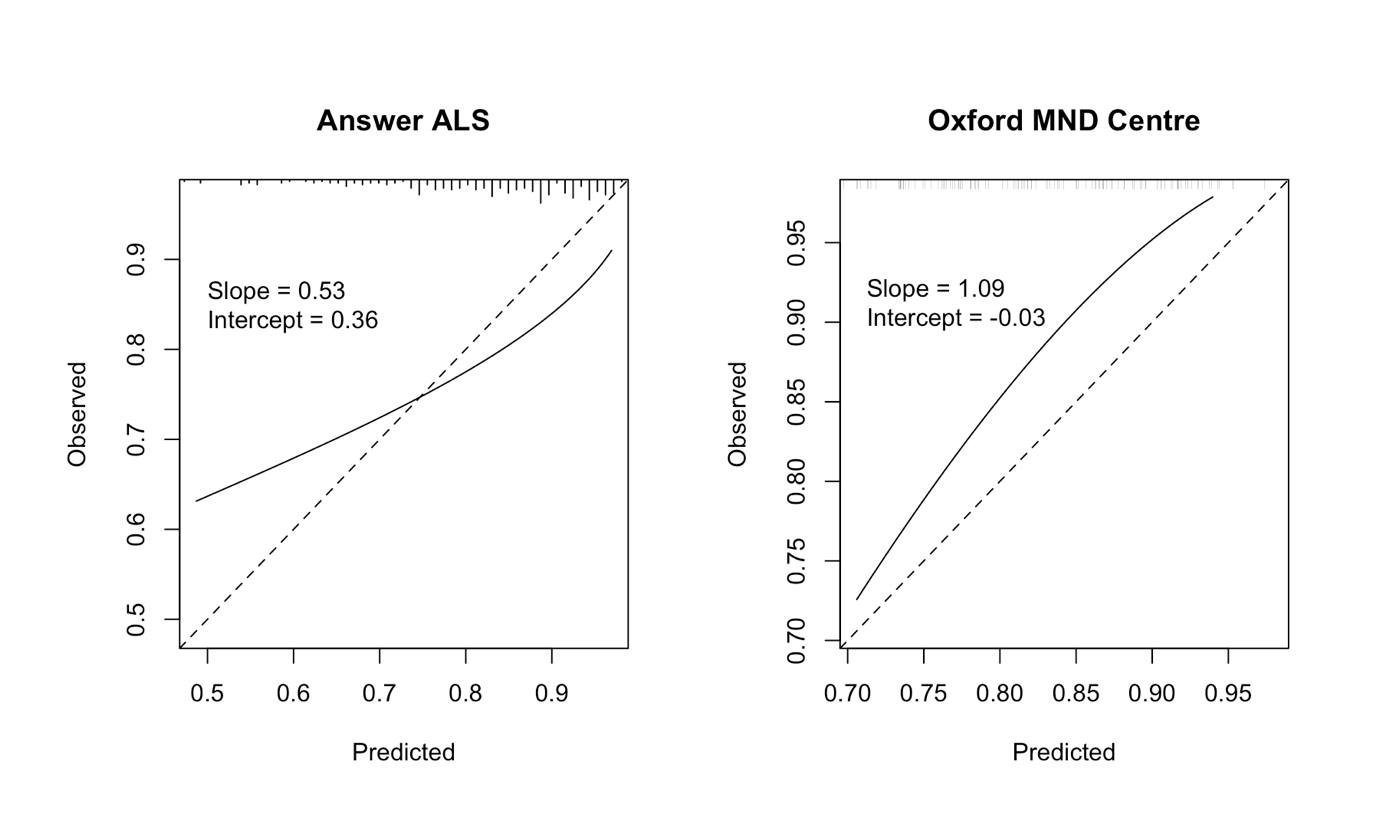
**Supplementary figure 4. Complete case-only sensitivity analysis overall calibration curves calculated using Cox-Snell residuals to evaluate the agreement between predicted and observed event rates, for (A) a Cox model trained in Oxford MND Centre data and tested in Answer ALS data, and (B) a Cox model trained in Answer ALS data and tested in Oxford MND Centre data. (C) and (D) show calibration plots, slopes and intercepts for one-year predictions using the same models as (A) and (B), respectively. ALS, amyotrophic lateral sclerosis; MND, motor neuron disease.
